# On The Quiet Power of National Decisions: Hospitals, State Aid, and Services of General Economic Interest

**DOI:** 10.1017/jme.2025.10146

**Published:** 2025

**Authors:** Mary Guy

**Affiliations:** School of Law and Social Justice, Liverpool John Moores University, United Kingdom

**Keywords:** Healthcare, Hospitals, State aid, Subsidy, Service of General Economic Interest

## Abstract

The extent to which EU competition law applies in the healthcare context remains a contested question. The contemporaneous publication of the European Commission’s Evaluation of State Subsidy rules for health and social services of general economic interest (SGEI) in December 2022, and of the Court of Justice of the European Union’s April 2023 judgment in Casa Regina Apostolorum regarding state support to hospitals in Italy, underscore uncertainty and appear to indicate an impasse. These publications unfold against the backdrop of two tensions: between state and market, and between the EU and national levels. Hospitals illustrate these tensions well due to the expansion of competition mechanisms into the hospital sector (notably expanding private provider delivery of public hospital services, often supported by “patient choice” policies), and of hospitals remaining typically local or regional in character rather than supranational. This article provides a timely and original analysis of the Casa Regina Apostolorum judgment in light of Commission policy regarding SGEI and hospitals and how EU Member States engage with this. It offers insights into the potential legacy of Casa Regina Apostolorum, and indicates where future legal challenges may focus.

## Introduction

In the ongoing — and evolving — discussions about the relationship between European Union (EU) competition law and health markets, hospitals may appear less topical than aspects such as pharmaceuticals,^1^ but nevertheless continue to occupy a distinctive place. This has recently been highlighted in the 2022 review^2^ by the European Commission (Commission) of the Services of General Economic Interest (SGEI) exception mechanism in healthcare (with hospitals given particular recognition), and the April 2023 judgment of the Court of Justice of the European Union (CJEU) in *Casa Regina Apostolorum*,^3^ a case concerning Italian state aid (which can be broadly equated to subsidies) and public hospitals in Italy. In general terms, SGEIs are services deemed essential for the public’s well-being and so are provided either by the state or by private entities under public service obligations. SGEIs can be designated in various sectors, including energy and transport, as well as healthcare and other social services.

The 2022 review (which focused on healthcare and social services) examined the operation of SGEI rules since 2012 including against the recent backdrop of the Russian war in Ukraine and the COVID-19 pandemic. While it broadly concluded that the rules remained “fit-for-purpose,” it also highlighted a call for further clarification of concepts relating to the applicability of EU competition law. The subsequent 2023 *Casa Regina Apostolorum* judgment appears to entrench a broad non-applicability of EU competition law in the healthcare context, and thereby perhaps raises as well as answers questions. An important connection between these two recent developments lies in what they can tell us about the approach at the EU level to *public* hospital activity and how this may provide wider learning about competition and healthcare.

The frequent coexistence of different types of hospitals within a country — which might broadly be categorized as public and private — and corresponding patient statuses and entitlements raise questions about the scope of markets, the benefits and limitations of competition, and the applicability of different aspects of competition law. In contrast to the US, where competition reforms in healthcare have been considered to prioritize efficiency over equity,^4^ the core solidarity basis of healthcare systems in Europe proves determinative for the scope of applying competition law.^5^ This links further to questions of the (appropriate) role of the state and markets in competition in healthcare at national and EU levels. In turn, this has led to modification of certain aspects of competition regulation (notably merger assessment), and non-application of other areas of competition law. Hospitals provide a particular example of where modifications of general competition regimes (notably merger control) have been deemed necessary in both the US^6^ and European countries.^7^

The distinctiveness of hospitals can be explained in part by the political sensitivities which can attach to them as distinct from, for example, health insurance companies, whose activities nevertheless pose important questions of healthcare access. The consistent specification and inclusion of hospitals in the Commission’s SGEI Decisions from 2005^8^ and 2012^9^ means specific attention is warranted in this present discussion, even though it is recognized that different classes of state aid cases can be identified — notably regarding risk equalization schemes — in connection with healthcare more broadly.^10^ Certainly the *BUPA* case^11^ — regarding risk equalization in Irish healthcare — has drawn attention^12^ to EU-level interest in competition in the healthcare context.

At the EU level, competition involving the hospital sector has been identified as one aspect of competition between healthcare providers which generates diverse views. For example, competition between hospitals has been cited as an example which may, or may not, yield better outcomes, leading to the importance of recognizing that “general conclusions are difficult to draw because the devil certainly is in the details.”^13^ These “details” might be deemed to include fundamental considerations such as the broad typology of healthcare systems across EU Member States, the coexistence of different types of hospitals (public, private not-for-profit, private for-profit, as well as mixed hospitals with both public and private offerings), and the corresponding patient group they treat (whether within or outside the public healthcare system). A key point is that across the 27 EU Member States, the healthcare sector varies from one Member State to another with regard to the financing scheme and the role of healthcare providers,^14^ suggesting that the sheer diversity would make a coherent EU-level approach to competition in the hospital sector difficult.

However, further complexities arise with the explicit engagement and applicability of EU law. Specifically, this involves a tension between the reservation of the healthcare system organization as a Member State competence under Treaty on the Functioning of the European Union (TFEU) Article 168(7)^15^ (as distinct from some greater scope for EU-level intervention in public health found within the broader Article 168), and how this national competence may be squared with applicability of EU competition law (whether antitrust or state aid). Applicability is defined by reference to the key functional concept of an “undertaking” i.e., “every entity engaged in an economic activity, regardless of the legal status of the entity and the way in which it is financed.”^16^ Member States also have scope to designate specific activities entailing a public service obligation as SGEI, and, broadly, the effect is that the EU competition rules then do not apply to these activities. Indeed, it appears possible for Member States to apply their national competition rules to the healthcare context even if EU competition law is not engaged — examples include the Netherlands and England (and also the UK while an EU Member State).^17^ The possibility of designating certain public service obligation-related activities as SGEI is thought to provide a serviceable exception to the antitrust rules in a social sector context,^18^ and by extension to the state aid rules in a healthcare context.^19^ Despite this, it has also been considered that the complexity of navigating the SGEI regime is such that countries may prefer to try to exempt their healthcare systems totally from the reach of the EU competition rules.^20^ Nevertheless, there is also evidence to suggest that the SGEI rules are seen as more accessible in view of the evolution of the Commission’s SGEI package.^21^

The foregoing discussion is intended to indicate that there are fundamentally two dynamics at play when considering competition in the hospital sector from the perspective of EU competition law: the tension between the state and the market, and the tension between the EU and Member State levels. This article engages with both aspects by considering first the context of EU healthcare systems and competition law before offering a timely analysis of both the evolution of state aid and hospitals cases culminating in the CJEU’s judgment in *Casa Regina Apostolorum*, and the aforementioned Commission’s 2022 review of the SGEI rules in the healthcare context. Where some discussions have considered whether the combination of SGEI and state aid decisions in the healthcare sector contribute to a more “Social Europe,”^22^ this contribution focuses on the dynamic between the EU and Member State levels in particular, and why this may become more determinative of the law in this area in the future. Finally, some proposals are made to help alleviate the perceived legal impasse which appears to have resulted from these recent developments.

## Framing the Interaction Between Competition, EU Competition Law, and Member State Healthcare Systems

The reach of EU law into national healthcare systems in general is a sensitive topic, particularly in view of perceptions of “overreach,”^23^ although the extent of this reach arguably varies between different aspects of EU law such as free movement of patients and competition law. A key consideration of how far EU competition law can penetrate national healthcare systems is TFEU article 168(7), which provides that healthcare system organization and health policy is a matter of national/Member State competence.

The power of TFEU article 168(7) to circumscribe the reach of EU competition law and facilitate the freedom of Member States to experiment with competition reforms in healthcare is, however, contested. A compelling narrative has developed whereby the applicability of EU competition law circumscribes this freedom,^24^ thus TFEU article 168(7) is presented in deference to TFEU article 107(1),^25^ which in general prohibits state aid to undertakings that distort competition, as seen in the Commission’s assessment of public funding to hospitals in Lazio (in what became the *Casa Regina Apostolorum* case).^26^ In contrast, it is also considered that TFEU article 168(7) represents a “delicate and sophisticated balance.”^27^ Indeed, even the Commission has indicated that the Estonian authorities’ choice to ensure adequate public healthcare services provided by hospitals by means of contracts concluded primarily with the public hospitals network “is covered by the Member States’ freedom under Article 168(7) TFEU to define its health policy and to organize the delivery of healthcare services and medical care. This choice does not in itself contravene the state aid prohibition of Article 107(1) TFEU.” ^28^ What is becoming clear in hospital state aid cases is that TFEU article 168(7) has received increasing levels of attention, including in Commission decisions relating to the hospital systems of Germany.^29^ The relevance of the national competence has been further underscored recently by Advocate General Pikamäe in the *Dôvera* case regarding health insurance in Slovakia, as determining that the applicability of the EU competition rules amounts to the EU courts “being asked to find a balance between the protection of undistorted competition on the internal market and respect for the powers of Member States.”^30^

The applicability of EU competition law — whether the “antitrust rules” (i.e., the prohibitions on anticompetitive agreements or abuse of dominance) or the prohibition on state aid — is triggered by the existence of an “undertaking,” defined as any entity engaged in “economic activity”^31^ which consists in offering goods and services on a market.^32^ Within this functional definition, a key consideration has been the emphasis on the nature of the activity, as distinct from, for example, the public/private status of the actor. Thus in *FENIN v Commission*,^33^ the state-funded status of managing bodies in the Spanish national health system were deemed not to act as “undertakings” when providing services free of charge to their members on the basis of universal cover or purchasing equipment in connection with this. FENIN had argued that these bodies acted as “undertakings” when they provided private care outside the Spanish public healthcare system and purchased equipment connected to this, but this distinction between healthcare purchasers and providers was rejected by the CJEU, on appeal from the EU General Court.^34^ Nonetheless, this purchaser/provider distinction has further been noted in the differing analyses of EU competition law cases in the healthcare context, with it being considered that the functional definition of an “undertaking” predominates in connection with healthcare providers, whereas more attention is paid to the wider healthcare context of the Member State and the role of solidarity in cases involving healthcare managing bodies/purchasers. These divergent approaches by the EU courts have been characterized as “abstract”/“concrete,”^35^ and in the specific context of state aid and hospitals, as “classic functional”/“attenuated functional.”^36^ A particular controversy regarding the purchaser/provider separation arose in the *FENIN* case,^37^ which saw the CJEU (in its fullest, Grand Chamber, formation) confirm that the “upstream” activity of purchasing activities did not displace the solidarity basis of healthcare provision within the Spanish taxation-funded public healthcare system. However, it was considered a missed opportunity to clarify the position of healthcare provision based on solidarity.^38^ Nevertheless, what had emerged prior to *Casa Regina Apostolorum*, was a consistent — if for some unsatisfactory — distinction: EU competition law was not deemed to apply with regard to healthcare purchasers (as reaffirmed recently by the CJEU’s 2020 judgment in *Dôvera* regarding Slovak health insurance),^39^ but was applicable to healthcare providers (following earlier cases such as *Pavlov*).^40^

The deceptively simple question of whether an entity is engaging in “economic activity” is tempered by the option for Member States to classify some activities as SGEI under TFEU article 106(2).^41^ An effect of this is to recognize that, within coexisting healthcare services, some activities may be subject to competition law, but others may not. A clear example is the distinction drawn in the *Ambulanz Glöckner* antitrust case^42^ between emergency ambulance services (SGEI) and standard patient transport (subject to competition law). The connection between the two suggests that existence of an “undertaking”/“economic activity” is key and the SGEI mechanism is parasitic on this, as demonstrated by the analysis in the Commission’s 2016 *Brussels Hospitals* analysis.^43^ This case highlighted the distinction between public sector and private sector delivery of public healthcare services being characterized by the additional obligation on public providers to ensure continuity.

**Figure 1.**
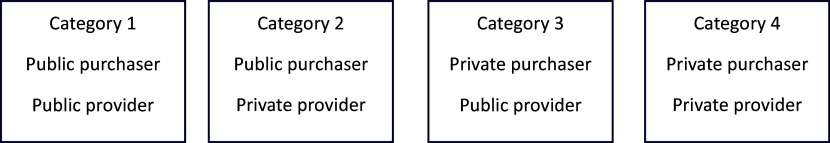
The “four categories” of European healthcare.

In order to understand some of the dynamics at play within national healthcare systems with regard to the applicability of EU competition law, it is helpful to consider how the interaction between public and private healthcare can be illustrated by four categories as shown in Figure 1.

In general terms, Category 1 activity may concern the provision of healthcare within a public healthcare system which may be paid for via taxation, or basic (as distinct from supplementary) insurance coverage. This may not fall within the scope of EU competition law insofar as there may be no relationship to which EU competition law can attach — the purchaser and provider may be one and the same entity.^44^ Category 1 activity may also be performed by a charity (such as a religious order), and may also fall outside the scope of EU competition law in the logic that the extent of public financing may prove determinative of the applicability of EU competition law. This logic characterized the finding of non-applicability of EU competition law in *Congregación de Escuelas Pías Provincia Betania v Ayuntamiento de Getafe*,^45^ which involved the delivery of education by a religious order.

At the other end of the scale, Category 4 represents a private healthcare market treating only private patients who pay for their treatment, and the full applicability of EU competition law to activities in this scenario appears uncontroversial.

Categories 2 and 3 have represented particular sticking points in analyses both of whether EU competition law applies in full with regard to hospitals, and whether the SGEI exception mechanism applies. This can be explained by the focus placed on solidarity as an underlying principle of EU Member State healthcare systems and the extent to which this can — or cannot — be said to be displaced by perceptions of the existence of competitive frameworks.

Category 3 activity can represent the situation where a patient may be required to pay for additional services within a public healthcare system, so where there may be private healthcare services delivered within public facilities. Such activities are thus distinct from the main activities of a public hospital because they are not typically based on principles of solidarity, nor universal in nature. For example, within the Estonian healthcare system, Category 3 activity may be considered to be represented by the Public Hospital Development Network Plan (HDNP) hospitals generating revenue beyond public funding via specified medical services, which are paid for by patients directly or by their private health insurance. This was examined in the context of the Commission’s Decision regarding alleged state aid to public HDNP hospitals, where the specified medical services included “treatments of uninsured persons and foreign patients, treatments of insured persons who choose to skip the waiting list, and occupational health services which are reimbursed by the employer.”^46^ In addition, it might be considered that Category 3 activity within the Estonian healthcare system might extend to “revenue from services delivered in the context of medical treatments,” such as “family wards and antenatal classes in the context of obstetric care, rehabilitation therapies where medical evidence is unclear (e.g., electrotherapy), vaccinations, patient transport, copies of records of radiological examinations,” and to “revenue from other side activities, notably customary amenities (staff canteens, renting out premises to small hospital shops, training-related revenue), and revenues from donations.” How such Category 3 activity factored into the Commission’s ultimate finding that competition law did not apply centered around the question of whether this activity was cross-subsidized by the public financing of the HDNP hospitals. Ultimately the Commission concluded that such cross-subsidization was not allowed, so that the Category 3 activities did not receive an advantage. This case is unusual as most of the hospital cases turn on what constitutes an undertaking.

Category 2 activity — whereby a patient is treated by a private provider, but the cost is absorbed by the public healthcare system — has proven the most contentious in case law thus far. Arguments in cases and academic literature^47^ have focused on the extent to which private providers deliver the same services as public providers, in the logic that if there is no difference, then a competitive market can be said to exist. A variation on this theme was tested in *Dôvera*, with the General Court suggesting that the existence of private health insurers converted the state insurer into an undertaking “by contagion,” although this logic was ultimately rejected by both Advocate General Pikamäe and the CJEU. A pattern which emerges from the case law, and has now been reconfirmed by *Casa Regina Apostolorum*, is that competition-related reforms such as the expansion of private sector delivery of public healthcare do not displace the underlying solidarity basis of a national healthcare system, so EU competition law may not apply.

Category 2 activity also provides a fertile ground for examining the scope of the SGEI mechanism, with considerations of the different roles played by public and private providers in underscoring conceptualizations such as a “genuine SGEI.” This was seen in the *Brussels Hospitals* ^48^ and *Klinikum Osnabrück* ^49^ cases, with differing obligations between public and private providers ensuring continuity of healthcare delivery.

The situation whereby an entity may deliver *both* public and private healthcare services (thus operating in Categories 2 *and* 4) was addressed to a certain extent in *PICFIC.* ^50^ This case saw PICFIC, an ecclesiastical, not-for-profit body which operated specialist clinics under the regime of “accreditamento” within the Italian public healthcare system (in Category 2) and for private patients (in Category 4) classified as an “undertaking,” thus subject to the EU state aid rules by virtue of the latter activity. The Commission took the view that “… at least as far as those [PICFIC] clinics provide healthcare services privately (at market prices and in competition with other private centers), they perform an economic activity and therefore qualify as an undertaking pursuant to Article 107(1) TFEU.”^51^ In so doing, the Commission introduced a level of nuance regarding how markets work in healthcare with the distinction being made between the various activities of hospitals (i.e., that some are economic, and some are not). This can become overlooked in findings which indicate discrepancies in the finding of “economic activities” in some hospital cases (such as *Brussels Hospitals*), but not others (such as *Casa Regina Apostolorum*).^52^ In other words, there is a clear distinction to be drawn between the behavior of essentially public hospitals, which can operate in Categories 1 and 3, and of private hospitals, whose primary focus may be Category 4, but nevertheless also include Category 2.

A final point to note in this overview of the applicability of EU competition law to hospitals is that while the core question of whether an “undertaking” exists in the healthcare context has received the most attention, there are further requirements to determine applicability of EU competition law — notably including an effect on trade between Member States. It is submitted that the local/regional/national character of a wide range of healthcare (and particularly hospital) activity is overlooked to an unhelpful degree in the EU-level context. Here a distinction between the antitrust and state aid rules emerges: whereas national equivalents of antitrust exist alongside the EU rules, state aid remains an exclusively EU-level regime with no national equivalent. This may seem to indicate that considerations of effects on inter-state trade should assume a different focus in the healthcare context. Indeed, the 2022 SGEI package review reiterated the importance of this aspect: “This notion is particularly relevant as, if there is no effect on trade, then there is also no State aid and the SGEI rules do not come into play.”^53^ This requirement for a cross-border “effect on trade” can be more or less straightforward to satisfy in various contexts, such as patient mobility in border regions between Member States.

From hospital state aid cases what emerges is that the inter-state trade effects attaching to hospitals can vary significantly, but at least two common themes may be considered to emerge from Commission reviews of hospitals in Czechia^54^ and Brussels.^55^

Firstly, the relevance of cross-border patient mobility. While this was found to be minimal in the Hradec Kralové region of Czechia (which shares a border with Poland), it proved determinative of an effect on inter-state trade in the context of Brussels hospitals, given their relative proximity to capital or at least larger cities in France, the Netherlands, and Germany, their multilingual status, and the fact that the Brussels Capital Region is home to a large number of citizens from other Member States. These juxtaposed findings offer a stark image, but nevertheless support broader findings that cross-border patient mobility in the EU is a “work-in-progress,”^56^ and that there may be good reasons to regard cities and regions (such as Brussels, Strasbourg, and Luxembourg) with a disproportionate emphasis on European citizenship (as distinct from national identity) as outliers for the purposes of establishing effects on inter-state trade in the EU competition law context.

Secondly, the presence of highly specialized hospitals with international reputations has been a factor in determining, respectively, the existence and lack of inter-state trade effects in the aforementioned Brussels and Czechia hospitals cases. This juxtaposition is again informative insofar as the existence of “highly specialized hospitals with international reputations” is likely to vary quite significantly across the hospital sectors of the Member States. While the “international reputation” aspect clearly speaks to the requirement for an effect on inter-state trade, the “highly specialized” characteristic relates more directly to the core consideration of solidarity within the healthcare system, and even to questions of whether a market can be said to exist.^57^ Thus while the need to access “highly specialized” care may be limited in terms of the numbers of patients accessing it, it nevertheless needs to be available to all, and not necessarily limited to a single country’s nationals.

These two aspects — the effect on inter-state trade and a highly specialized focus — also featured in the aforementioned PICFIC case. While the liquidity of PICFIC was linked with its specialist status as noted above, it is also interesting to note that the Commission also acknowledged the scope for inter-state trade effects arising from PICFIC’s Category 4 activities:^58^

“By granting PICFIC access to liquidity at conditions which it would not otherwise obtain, the State guarantee is liable to improve the competitive position of the centres it operates in relation to its competitors in the internal market. It consequently distorts or threatens competition and affects trade between Member States.”

This review of hospital state aid cases has demonstrated the significance of the distinction between private providers delivering services within the public healthcare system (Category 2 activity) and for private patients (Category 4 activity) in triggering the applicability of EU competition law, as demonstrated by juxtaposing *PICFIC* with cases such as *Estonian Hospitals.* Furthermore, the review has not only highlighted the increased acknowledgement of Member State competence with regard to national healthcare system organization under TFEU article 168(7), but also the importance of effects of inter-state trade, which can be illustrated by cross-border patient mobility (*Czechia Hospitals*) and/or the international reputation which can attach to highly specialized hospitals (*Brussels Hospitals*). It is against this backdrop that the *Casa Regina Apostolorum* case is now examined.

## State Aid and Hospitals Cases: Does *Casa Regina Apostolorum* Represent an Impasse, or Just a Change of Focus for the EU Level Regarding Competition and Healthcare?

Casa Regina Apostolorum is a religious congregation that owns a private hospital that delivers healthcare services in the Lazio region. It made an allegation of illegal state aid being provided to public hospitals in the same region. More specifically, it argued that public funds paid to public hospitals operating within the Italian public healthcare system (SSN) to cover their financial deficits without verification of their costs would be in breach of the principles of patient choice and competition to the detriment of private hospitals which also delivered SSN services.^59^ The complainant went on to argue that reforms enacted in Italian healthcare, notably the conversion of public hospitals into corporations subject to managerial principles, and the underpinning of the SSN on the principle of “patient choice” “… would have introduced competition into the SSN system and made the services economic in nature.”^60^

Thus far, the facts of *Casa Regina Apostolorum* indicate the existence of a Category 2 situation, posing the question of the extent to which competition reforms (specifically policies enabling patient choice of public or private provider) may be said to displace the underlying solidarity basis of the Italian healthcare system. The complainant sought to strengthen its argument in this regard by indicating Category 3 activity by the public hospitals, specifically delivering private healthcare services within the *attività libero professionale intramuraria* (ALPI) system.^61^ A combination of these factors would have implications for overcompensation of the public hospitals (by virtue of their private healthcare activities) and under-compensation of the private hospitals (by virtue of their public healthcare activities).^62^

The Commission found that the solidarity basis of the Italian healthcare system was neither displaced by the development of patient choice policies and other competition reforms, nor challenged by the public hospital’s delivery of private healthcare services. By combining Category 2 and Category 3 activities, it concluded that the EU state aid rules did not apply. The Commission’s logic in this conclusion was based in part on the definitions of solidarity which emerged from previous case law (notably *FENIN* ^63^ and *AOK Bundesverband*),^64^ but also on its characterization of the Italian healthcare system as solidarity-based from the 2012 *ICI-IMU* state aid case.^65^ While this latter case is concerned with exemptions from municipal taxes relating to real estate used by noncommercial entities, it nevertheless afforded the Commission an opportunity to review the Italian healthcare system. The Commission concluded that the entities in that case did not qualify as undertakings due to characteristics of the Italian public healthcare system such as the provision of universal cover, solidarity basis, the direct funding of public hospitals from social security contributions and other state resources, and the fact that public hospitals provide their services free of charge on the basis of universal cover or for a low fee which covers only a small fraction of the actual cost of service.^66^

The continuation of the combined logic of *AOK Bundesverband* and *FENIN* to exclude applicability of the state aid rules in the context of *Casa Regina Apostolorum* is arguably not surprising in light of a sense of consistency emerging across the specific category of hospital cases: thus, similar arguments have been advanced in connection with the aforementioned Estonian hospitals case. The Commission’s finding in *Casa Regina Apostolorum* is also not surprising in light of its previous finding in *ICI-IMU* where it indicated the solidarity basis of the Italian healthcare system (although it did not examine the activities of hospitals in this case). While the Commission’s finding in *PICFIC* — which was concerned with clinics operating within the Italian healthcare system — appears contradictory if “healthcare” is understood broadly (with no distinction between Categories 2 and 4), this is undermined by the fact that PICFIC’s private healthcare activities (i.e., Category 4) appeared key to determining applicability of the state aid rules (with the Commission remaining silent on Category 2 activities). It should further be noted that the resulting state aid for PICFIC was permitted in connection with guidelines on rescuing and restructuring which, in conjunction with the wider state aid assessment criteria, enabled due attention to be paid to the specialist nature of services provided by PICFIC, its important status at national and regional levels, and accordingly the difficulty of substitution by other providers.^67^

Further to the Commission’s finding that the state aid rules did not apply in *Casa Regina Apostolorum*, the complainant appealed first to the General Court, and latterly to the CJEU, advancing arguments, inter alia, regarding SGEI in the Italian healthcare system which are examined in the next section. The fact that the General Court and the CJEU both upheld the Commission’s finding may not be surprising — particularly in view of the entrenchment of the view that degrees of competition are insufficient to displace the solidarity basis of a healthcare system seen in the CJEU’s 2020 ruling in *Dôvera* regarding Slovak health insurance. Nevertheless, the CJEU’s finding in *Casa Regina Apostolorum* is striking for at least two reasons.

First, because it sees the “concrete” (solidarity) approach being applied to healthcare *providers* for the first time before the CJEU.^68^ As indicated above, this approach — of examining the wider national healthcare context and solidarity basis, as distinct from the more “abstract” or functional interpretation of an “economic activity” — has developed across a range of cases involving healthcare managing bodies (purchasers). This distinction — effectively between purchasers and providers — which can underpin competition reforms in healthcare created a curious discrepancy, which was arguably exemplified by the *FENIN* case, which concluded that the “downstream” activity of healthcare *provision* being solidarity-based in the taxation-funded Spanish healthcare system outweighed suggestions that the “upstream” activity of *purchasing* could be separated from this and considered economic. In *FENIN*, the lack of explicit attention to the status of purchasers within a solidarity-based system and EU competition law led to this question being left open.^69^ As such, resolution could have taken one of two forms: Either managing bodies/purchasers would subsequently be found to be “undertakings,” or providers would be confirmed not to be “undertakings.” The former might be inferred by the General Court’s judgment in *Dôvera*, with the aforementioned suggestion that the state health insurer would “by contagion” be regarded as an undertaking alongside the private insurers. However, this was ultimately dismissed by the CJEU.

Secondly, the absence of an Advocate General Opinion^70^ in *Casa Regina Apostolorum* means that various questions remain unanswered between the General Court and CJEU judgments. However, it further arguably indicates most fundamentally that there was no new question of law to be interpreted in the case. This makes sense in view of the Commission (and the EU courts generally) perhaps seeking consistency with earlier decisions. It is noted that Advocate General Pikamäe — who had delivered an Opinion in *Dôvera* — appeared nevertheless assigned to *Casa Regina Apostolorum* but (disappointingly, if not unusually) did not issue an Opinion. Without presuming to speculate what an Opinion in *Casa Regina Apostolorum* may have looked like, it is nevertheless possible to see how some of the same general points could have relevance in both cases. For example, the possibility of overestimating the impact of the degree of competition permitted within the Italian healthcare system, and the role of competition relating to management (as per *AOK Bundesverband*) rather than being transformative of the underlying solidarity basis per se. This may help understand why an Opinion appeared not to be deemed necessary in *Casa Regina Apostolorum.*

Thirdly, the finding of non-applicability of the state aid rules to healthcare providers in *Casa Regina Apostolorum* appears to create an impasse insofar as it is difficult to see how future allegations of illegal state aid in the healthcare (and specifically hospital) context may be formulated. Indeed, the CJEU’s judgment has prompted the question of whether the EU level has somehow lost interest in competition in healthcare.^71^ While this question is significant, it can also be considered that an effect of the CJEU’s judgment will be to refocus competition in healthcare to the national level such that cases will be (still more) rare than *Dôvera* and *Casa Regina Apostolorum.* The emphasis placed on TFEU article 168(7) and the determination of solidarity as a basis for a healthcare system as being a matter for national determination could lead to inferences that future EU-level interest in healthcare may be reignited only where there is a clear sense of scope for anticompetitive conduct with effects which transcend national boundaries. This arguably requires more explicit analysis of the parameters of the internal market in the healthcare context: It may be the case that there are some regions which give rise to more concerns than others about inter-state effects on trade, which may be due to a particular level of patient mobility, or with regard to a particular specialist hospital service enjoying an international reputation. Put simply, neither of these aspects were in evidence in *Casa Regina Apostolorum*, where the hospital services appeared more standard, and were located in Lazio, a region which, while accommodating both the capital city and the Vatican, would not seem to offer clear scope for cross-border patient mobility.

The focus on hospital services in *Casa Regina Apostolorum* may yet prove determinative for the legacy of this case insofar as “healthcare” may be too broad a categorization, and a need for a more “granular” approach may yet emerge as beneficial in disaggregating different aspects of healthcare delivery. However, it is acknowledged that *Dôvera* signaled a reinforcement of a lack of EU-level interest in competition with regard to purchasing activities. Certainly, the link with the SGEI mechanism (discussed below) may become more, rather than less, prominent. Alternatively, the EU-level focus may shift to Category 4 activity among private providers, rather than Category 2 activity. Thus, the Category 4 activity found in the aforementioned *PICFIC* case may prove to be more of a focus for EU-level interest, given that this case saw the alleged aid justified under TFEU article 107(3)(c)^72^ rather than be exempted at the hurdle of an “undertaking.”

A final key takeaway from *Casa Regina Apostolorum* is not that competition in healthcare cannot be of interest, or deemed not problematic, but rather simply that it may not be a matter for EU-level focus. Certainly, where there are competition reforms at a national level, there is evidence to show that amendments to national law may diverge from EU case law. This was seen with Article 122 of the Dutch Health Insurance Act 2006 (*Zorgverzekeringswet*), which enabled private health insurers to be subject to Dutch competition law following clarification of the limits of EU competition law by the CJEU’s 2004 judgment in *AOK Bundesverband* regarding German sickness funds.^73^ Prior to the 2006 reforms, the Dutch healthcare system had also relied on sickness funds (*ziekenfondsen*), but the incorporation of private health insurers was seen as requiring the option of applying at least national competition law to support the then new focus on competition.

## SGEI Decision and Hospitals Beyond the 2022 SGEI Package Review — Where Now?

A pressing question concerns the implications of the CJEU’s April 2023 *Casa Regina Apostolorum* judgment in light of the Commission’s 2022 SGEI package review. This was published in December 2022 and had explicitly called for further clarification of the intrinsic distinction between “economic” and “non-economic” activities in light of the CJEU’s 2020 *Dôvera* judgment and the General Court’s 2021 judgment in *Casa Regina Apostolorum.* On the face of it, the lack of “economic activities” in healthcare following *Casa Regina Apostolorum* and *Dôvera* might seem to undermine recourse to the SGEI decision. Indeed it had already been indicated (in a discussion referencing the context of Slovenian healthcare reforms) that national governments may prefer to exempt healthcare systems totally rather than engage with the complexity of the SGEI mechanism.^74^ However, the Commission’s 2022 review of the SGEI package broadly concluded that the SGEI package remained essentially “fit for purpose,” subject to the aforementioned requirement for further clarifications.

### Hospitals, Healthcare, and the SGEI Package

The SGEI package, initially introduced in 2005 and updated in 2012, comprises a range of legal instruments which seek to reduce the administrative burden on Member States’ compliance with the EU state aid rules with regard to public service compensation. The most important of these instruments is the Decision, which in both iterations since 2005 has clearly specified hospitals as a candidate to benefit from reduced requirements in recognition, inter alia, of the potential need for compensation which may exceed specified thresholds. In the 2012 Decision, “social services,” which include “health and long-term care services” were specified in addition to hospitals. The SGEI package review of 2022 would seem to indicate that the focus on both these health-related categories would be likely to continue.

The rationale for specifying hospitals in the 2005 Decision was set out in Recital 16:^75^

“Hospitals … which are entrusted with tasks involving services of general economic interest have specific characteristics that need to be taken into consideration. In particular, account should be taken of the fact that at the current stage of development of the internal market, the intensity of distortion of competition in those sectors is not necessarily proportionate to the level of turnover and compensation. Accordingly, hospitals providing medical care, including, where applicable, emergency services and ancillary services directly related to the main activities, notably in the field of research, … should benefit from the exemption from notification provided for in this Decision, even if the amount of compensation they receive exceeds the thresholds laid down in this Decision, if the services performed are qualified as services of general economic interest by the Member States.”

The rationale for including hospitals and “social services” in the 2012 Decision was couched in broadly similar terms in Rationale 11, albeit with the further recognition that “A larger amount of compensation for social services [with hospitals referenced by analogy] does thus not necessarily produce a greater risk of distortions of competition.”^76^

What might be inferred from this is that there was an anticipation that as national healthcare systems sought to increase interactions between public and private healthcare, this could translate to effects at EU internal market level. Whether this has happened is moot, given the various nuances between national healthcare systems and the extent to which competition has developed. It is, however, interesting to note that research conducted in the context of the 2022 SGEI package review indicates that evolution of competition in the healthcare market is influenced by factors as diverse as hospital spending, the variation in hospital numbers, and particularly the coexistence in selected Member States of public, not-for-profit, and private for-profit hospitals being deemed not to necessarily lead to competition between these actors due to differences in the services they provide.^77^

The recognition of hospitals in particular as somehow “special” and deserving of particular treatment arguably fits well with the narrative of the SGEI package more generally affording recognition of the importance of democracy and indeed deference to the national level in the provision of public services.^78^ This is logical for hospitals, which attract a particular degree of political sensitivity relative to other aspects of healthcare. Thus, it has been considered that politicians will campaign against local hospital closures, and this will likely attract more media attention than, say, a merger of two health insurance companies, even if this ultimately has implications for healthcare access.

However, the SGEI mechanism places a certain degree of responsibility on the Member States in designating particular activities as SGEI — the Commission’s role is essentially to review the national decision for concerns of manifest error. Logically this would suggest that a diversity of SGEI will be identified across the Member States given the national competence attaching to healthcare system organization under TFEU article 168(7).

Indeed, this diversity is illustrated by the annual SGEI reports^79^ which Member States submit to the Commission in conjunction with the SGEI package. In Austria, for example, various rescue services are typically designated SGEI, including the public rescue service (professional transport of patients), civil protection (implementation of disaster prevention and control), and rescue organizations for water rescue and mountain rescue services. Other countries include more or less detailed breakdowns of the nature of treatments which may be included within the context of SGEI seemingly across the whole country (e.g., Czechia, Belgium, Germany, Latvia, Luxembourg, Slovakia), while others are confined to specific regions, such as Asturias in Spain. In the Netherlands, where there has been active engagement with the EU law framework in view of competition reforms within the national healthcare system, recent examples of SGEI include the organization of organ and tissue donation to respond to donor shortage (2020–2021), and antibiotics policies (2018–2019). In contrast, reports from Denmark indicate a consistent rejection of the SGEI mechanism and indeed the state aid rules as applicable to the Danish healthcare system: “It is the opinion of the Danish authorities that the financing of public hospitals in Denmark concerns services that cannot be regarded as covered by the State aid rules in Article 107.”

This diversity of the scope for SGEI in national healthcare systems illustrates well the logic for having SGEI determination as a national competence. Indeed, in its 2022 SGEI review, the Commission itself emphasized that

“While the [state aid and SGEI] rules set out how the Commission will assess aid measures and allow Member States to grant support, they do not oblige Member States to grant aid; this remains in their discretion. Indeed, Member States are free to choose other policy instruments to reach a certain goal.”^80^

While calls have been made for further EU-level clarification to assist Member States,^81^ the Commission’s 2022 SGEI review acknowledged that further prescriptiveness may be unhelpful:^82^

“Since the distinction between economic and non-economic activities depends to some extent on political choices and economic developments in a given Member State, it is not possible to draw up an exhaustive list of activities that a priori would never be economic. Such a list would not provide genuine legal certainty and would thus be of little use.”

### What Is the Connection Between the SGEI Decision and the State Aid Rules?

It was suggested above that there is a prima facie connection between the SGEI Decision and the state aid rules. This has been borne out, inter alia, by the extensive *Brussels Hospitals* case. While this case was ostensibly concerned with clarifying the extent of the Commission’s role in examining “manifest error” in Member State definition of SGEI, it nevertheless reiterated the establishment of an “economic activity” under Article 107(1) TFEU as a necessary prerequisite to reviewing recourse to the SGEI mechanism under Article 106(2) TFEU which provides the legal basis for the SGEI package.

However, other hospital cases suggest that the connection between the SGEI Decision and the state aid rules is less clear. This can be explained by use of the “block exemption” approach of the SGEI Decision in the aforementioned *Klinikum Osnabrück* and *Czechia Hospitals* cases.

In view of these considerations, the specification of hospitals in the SGEI Decision appears curious. One explanation may lie in the establishment of state aid under TFEU article 107(1), and the test for SGEI exemption under TFEU article 106(2) as being sequential, but ultimately distinct with different aims.^83^ This distinction has been reformulated as two questions: firstly, whether there is an aid, because an advantage is conferred; secondly, if this is so, whether the aid should be exempted.^84^ Whether these questions can — and indeed should in specific instances — displace the overarching question of whether an “economic activity” exists then takes on a particular significance.

To do so would lend support to the view that the SGEI package for social services operates in the state aid context by analogy with block exemptions in antitrust.^85^ Block exemptions have been considered to “automatically discharge certain categories of agreements from the EU prohibition on anticompetitive agreements without engaging in a case-by-case analysis,” and thus offer “a legitimate and effective tool for the consideration of public policy.”^86^ From the perspective of state aid, the repeated specification of hospitals in the SGEI package generates two insights. Firstly, that this specification may be sufficient to obviate circular re-examinations of whether hospital-related Category 2 activity amounts to an “economic activity,” and thus falls within the scope of the prohibition on state aid of TFEU article 107(1). This is supported by findings of the Commission that the SGEI Decision applies: “In light of this, the Commission does not analyze whether the cumulative conditions of State aid within the meaning of Article 107(1) TFEU are fulfilled” in the *Klinikum Osnabrück* case, and “… for the sake of completeness, the Commission observes that those measures would in any event be block exempted and therefore compatible with the internal market” in the *Czechia Hospitals* case.

Secondly, and relatedly, that there is still scope for EU-level review of national decisions to classify particular activities as SGEI, given the long-standing recognition^87^ that the Commission’s “competence is limited to checking whether the Member State has made a manifest error when defining the service as an SGEI.” The extent of the Commission’s role in this regard was explored in the *Brussels Hospitals* case.

A final consideration in this regard is *how* the SGEI mechanism is raised in state aid cases, and *where* a TFEU article 107(1) review may be favored. For example, in cases such as *Klinikum Osnabrück* and *Czechia Hospitals*, it was, respectively, the German and Czech authorities who raised engagement with the SGEI mechanism. In contrast, the SGEI mechanism appears not to be raised in the *Estonian Hospitals* case, and so a full TFEU article 107(1) review was conducted with near-exclusive emphasis on the “undertaking” question.

What emerges from this is that while a complainant’s allegation of illegal state aid typically starts from the presumption that the respondent’s activity is economic, the Commission’s investigation may be tempered by one of two possible responses by the Member State authorities. First, where national authorities advance no engagement with SGEI (as seen in the *Estonian Hospitals* and *Casa Regina Apostolorum* cases), this may lead the Commission to conduct a “full” TFEU article 107(1) analysis which engages with the question of whether or not there is an “economic activity” by reference to the national context (thus following the aforementioned “concrete” approach”^88^ or “attenuated functional” approach).^89^ The second response indicates that the national authorities have engaged with the SGEI mechanism (as seen in the *Klinikum Osnabrück* and *Czech Hospitals* cases), in which case the Commission’s line of inquiry may shift to the block exemption of the 2012 SGEI decision and whether the associated requirements have been complied with. This discrete question further raises the possibility of a further challenge on the basis of the Commission’s scope to investigate whether a Member State has made a “manifest error” in defining SGEI. Whether this apparent practice of the Commission can continue in light of more recent (2020^90^ and 2024^91^) CJEU judgments confirming the need to establish the existence of state aid as a first step is moot. However, the special recognition accorded to healthcare and hospitals may be sufficient to justify this.

Despite this seeming encouragement for Member States to engage with SGEI, it was noted above that there appears a reluctance to do so in view of the technical aspects of the mechanism.^92^ The Conclusions of the 2022 SGEI review’s call for greater clarification of concepts may help in this regard. A further, related, initiative to the SGEI package is a new requirement in the 2023 SGEI de minimis Regulation^93^ for information on de minimis aid granted for SGEI to be entered in a central register at national or EU level from January 1, 2026.

### Casa Regina Apostolorum and SGEI

In *Casa Regina Apostolorum*, it is important to note that reference to SGEI was initiated by the complainant, rather than the Italian authorities. The complainant attempted to support their assertion of an economic activity (and thus unlawful state aid) by reference to SGEI in the healthcare context being identified in Italy’s SGEI reports. The Commission rejected this:^94^

“The Commission points out that the reporting of certain activities in the SGEI reports is not evidence of the economic nature of the activities. In any event, the Italian authorities have explained that the activities were included in the reports to describe better to the Commission the nature and workings of the SSN. Furthermore, within the SGEI Report for years 2012–2013 the Italian authorities explicitly explained that the organisation of the SSN does not fall within the scope of the SGEI rules.”

These appear to have contributed to further claims by the complainant, for example in the request that the General Court engage with considerations regarding SGEI and the evolution of the Italian authorities’ conception of this.^95^ The General Court declined to do so, pointing out that the Commission could not be expected to do this given the finding of no economic activities, although it recognized that this was a separate matter from the merit of the complainant’s assertions.^96^ In its judgment, the CJEU reaffirmed the correctness of the Commission’s approach.^97^

What might be inferred from the Commission’s comment that reporting certain activities is not evidence of their economic nature is not that the reports amount to an “admission,” or that Member States lack competence in defining an “economic activity” (although this is an EU-level concept). Rather, the comment is instructive for implicitly underscoring the decoupling of SGEI and the TFEU article 107(1) questions.

## Concluding Remarks

This paper has explored how hospitals provide a unique lens for considering how the EU’s state aid rules can operate in connection with the healthcare context more broadly. It started from the premise that an unsatisfactory impasse appeared to emerge between the 2022 SGEI review and the 2023 *Casa Regina Apostolorum* judgment. By drawing on this, as well as other cases typically focused on hospitals, and the hospital focus of the SGEI decision, at least four insights emerge.

First, that by outlining the “four categories of European healthcare,” it becomes possible to systematize — in broad terms — different interactions between coexisting public healthcare systems and private healthcare markets. This helps explain perceived inconsistencies between cases. Notably, by juxtaposing *PICFIC* and *Casa Regina Apostolorum*, it becomes possible to see how the EU level may have more interest in private healthcare provider activity on the private healthcare market in Category 4 (which may have an effect on inter-state trade) rather than the activity of public healthcare providers in general terms.

Secondly, by a particular focus on hospitals, it becomes clear that determining the economic nature of an activity is only part of a wider puzzle of how the EU state aid rules (and competition law more generally) may operate in connection with healthcare markets. Given the typically regional or national focus of hospitals, it becomes clear that there may be considerably less scope for wider effects on the internal market via considerations such as cross-border patient mobility in specific areas, but that international specialization may also factor into EU-level assessments.

Thirdly, the discussion reaffirms that Member States have two wide discretions: under TFEU article 168(7) with regard to healthcare system organization, and in defining SGEI. The former may be circumscribed by questions of applicability of the state aid rules, but decisions such as *Dôvera* and *Casa Regina Apostolorum* appear nonetheless to reinforce this discretion under TFEU article 168(7). Furthermore, the latter discretion of defining SGEI may also ultimately not be fettered, or even overridden, by the “undertaking” question. This appears supported by the discrepancy which emerges between those hospital cases in which the Member State authorities raise their engagement with SGEI (e.g. in the *Czechia Hospitals* and *Klinikum Osnabrück* cases), and those where they appear not to (e.g. *Estonian Hospitals*). The latter category may now be heavily constrained by the CJEU judgment in *Casa Regina Apostolorum.*

Fourthly, it might be considered that the EU-level interest in competition in national healthcare systems is simply refocused by the *Casa Regina Apostolorum* judgment, particularly given the application of the “concrete” approach now to providers as well as managing bodies (following *Dôvera*). As noted above, the development of competition reforms in healthcare can be addressed as a national matter. The experience of the Dutch government in enacting legislation to distinguish the applicability of Dutch competition law to private health insurers from the EU-level finding of non-applicability of EU competition law to German sickness funds can prove instructive here.

Finally, these clarified parameters of EU-level assessments could encourage future cases to engage with questions of whether “manifest error” exists in connection with Member State definition of SGEI. Indeed, the situation in *Casa Regina Apostolorum* could arguably be couched in these terms, and the Italian authorities’ evolving conception of SGEI explored in this light. Perhaps a renewed engagement with the SGEI Decision by Member States can be the real legacy of *Casa Regina Apostolorum*, along with greater recognition of the key role played by the national competence of Member States with regard to healthcare. Certainly, *Casa Regina Apostolorum* has provided some of the clarification sought by the 2022 SGEI review by clarifying the non-applicability of EU competition law to healthcare providers. However, undoubtedly more is needed.
